# A priori estimation of sequencing effort in complex microbial metatranscriptomes

**DOI:** 10.1002/ece3.6941

**Published:** 2020-11-05

**Authors:** Toni Monleon‐Getino, Jorge Frias‐Lopez

**Affiliations:** ^1^ Section of Statistics (Department of Genetics, Microbiology, and Statistics) University of Barcelona Barcelona Spain; ^2^ BIOST^3^ GRBIO (Research Group in Biostatistics and Bioinformatics) Barcelona Spain; ^3^ College of Dentistry University of Florida Gainesville FL USA

**Keywords:** machine learning, metagenomics, metatranscriptomics, NGS, rarefaction curve, sample size, sequencing effort, simulation

## Abstract

Metatranscriptome analysis or the analysis of the expression profiles of whole microbial communities has the additional challenge of dealing with a complex system with dozens of different organisms expressing genes simultaneously. An underlying issue for virtually all metatranscriptomic sequencing experiments is how to allocate the limited sequencing budget while guaranteeing that the libraries have sufficient depth to cover the breadth of expression of the community. Estimating the required sequencing depth to effectively sample the target metatranscriptome using RNA‐seq is an essential first step to obtain robust results in subsequent analysis and to avoid overexpansion, once the information contained in the library reaches saturation. Here, we present a method to calculate the sequencing effort using a simulated series of metatranscriptomic/metagenomic matrices. This method is based on an extrapolation rarefaction curve using a Weibull growth model to estimate the maximum number of observed genes as a function of sequencing depth. This approach allowed us to compute the effort at different confidence intervals and to obtain an approximate a priori effort based on an initial fraction of sequences. The analytical pipeline presented here may be successfully used for the in‐depth and time‐effective characterization of complex microbial communities, representing a useful tool for the microbiome research community.

## Graphical abstract

New method to calculate the effort in saturation curves and a priori genes prediction using a simulated series of metatranscriptomic/metagenomic matrices.

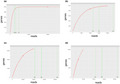

## 1 INTRODUCTION

The study of the human microbiome has dramatically expanded our understanding of the role that microbes play in health and disease. These studies have been facilitated by the development of next‐generation sequencing (NGS) technologies, which are capable of generating enough sequences to cover most of the diversity present in a sample. However, capturing the full composition is still a challenge, even when estimated by the distribution of 16S rDNA sequences (Ni et al., 2013; Tamames et al., 2012). The study of transcriptomes of whole microbial communities, or metatranscriptomics, has increased exponentially in the last few years (Shakya et al., 2019; Zhang et al., 2019), and trying to analyze these kind of data has produced a new set of challenges. Thus, García‐Ortega and Martínez (2015), using a nonparametric estimator for the number of undetected genes, found that on average, approximately 10% of the expressed genes per accession remain undetected if individual sequencing libraries are analyzed. The power and accuracy of such experiments depend substantially on the number of reads sequenced, so a crucial step in the experiment design should be determining the optimal read depth for a particular study or verifying whether the experiment has adequate depth (Robinson & Storey, 2014).

Most RNA‐seq studies have focused on assessing the depth of transcriptome sequencing in eukaryotic systems, using a wide range of estimated sequencing depths to cover the full patterns of expression. In the human transcriptome, the sequencing depth estimated as necessary to observe differences in expression profiles varies from 100 to 700 million sequences (Toung et al., 2011; Westermann et al., 2012). In the case of prokaryotic RNA‐seq experiments, Haas et al. have shown that the reads typically produced in a single lane of the Illumina HiSeq sequencer far exceed the number needed to saturate the annotated transcriptomes of diverse bacteria growing in monoculture (Haas et al., 2012).

In metatranscriptome sequencing, saturation is reached when an increment in the number of reads does not result in an additional increment in the number of expressed transcripts, or no additional ORFs are detected in the case of shotgun metagenomic analysis. One way of estimating the point of saturation is by using rarefaction curves, a method commonly used in ecology to estimate the species richness as a function of sampling effort. In the case of RNA‐seq/DNA‐seq, a higher sequencing depth will only prolong the curve but is otherwise comparable to a lower sequencing depth curve for the same regions. Once the curve reaches a plateau, where additional sequencing would only marginally increase the number of transcripts observed, the curve can be considered as saturated, and there is therefore no need to increase the sequencing effort to describe the gene expression profiles of the community. Another useful feature of saturation curves is that they allow the complexity of the sample to be assessed: Expressed transcripts will be numerous in highly complex communities and low in those with low complexity.

We have developed a method to calculate the sequencing effort needed to reach the maximum number of existing genes using rarefaction curves extrapolating from a small initial sequencing depth (10%–20%) and estimating the confidence intervals at 90%, 95%, or 99% of the maximum sequencing effort.

## 2 MATERIAL AND METHODS

### 2.1 Methodological overview

We first simulated more than a thousand different metatranscriptomic/metagenomic matrices. On those matrices, we computed rarefaction curves using the function iNEXT( ) from the iNEXT R library for Interpolation and Extrapolation for Species Diversity (Hsieh et al., 2016). We then used a nonlinear growth model to compute the maximum number of genes expected and to estimate the sequencing depth (reads) required for 90%, 95% or 99% of the maximum sampling effort.

Finally, using a method based on machine learning, we predicted the 90%, 95%, or 99% of the maximum number of genes using only a minimum number of sequencing depth (reads) and the sampling effort needed. All these functionalities were included in some functions of R. The method was tested, as an application thereof, on metatranscriptomic samples of an oral microbiome. The results are presented in the supplementary material of this article.

### 2.2 Simulation of metatranscriptomic/metagenomic matrices

Metatranscriptomic/metagenomics matrices were simulated as described in Rodríguez‐Casado et al. (2017) and in Monleon‐Getino et al. (2019). In order to simulate gene data and the associated reads in each sample, it is necessary to know which underlying probability model best explains the distribution of the data, for example, the binomial (distribution of the reads per gene in a sample), multinomial (distribution of the counts for the set of genes in the sample), and complex distributions such as the Dirichlet‐multinomial (distribution of reads for the set of genes and samples in the experiment). The following is a brief theoretical introduction to these distributions, which once known allow the simulation of new samples by Monte‐Carlo simulation, a statistical method used to solve complex mathematical problems through the generation of random variables.

Table 1 shows the general metatranscriptomic/metagenomic matrix (***M***) structure (*n* rows: samples, *p* columns: genes) obtained after the bioinformatic analysis, which constitutes the starting point of this study. Outlined below are the mathematical formalization and the study of probability distribution, previously studied in more depth (Monleon‐Getino et al., 2019).

**TABLE 1:** Data matrix structure of ***M***′ (metatranscriptomics or metagenomics matrix input)

Number	Gene	Sample 1	Sample 2	Sample *jth*	Sample *n*	Total
1	type. 1	*m_11_*	*m_12_*	*…*	*m_1n_*	$N_{1.}$
2	type. 2	*m_21_*	*m_22_*	*…*	*m_2n_*	$N_{2.}$
⋮	⋮	*…*	*…*	*m_ij_*	*…*	*…*
*k*	type. k	*m_k1_*	*m_k2_*	*…*	*m_kn_*	$N_{k.}$
	Total	$N_{.1}$	$N_{.2}$	…	$N_{.n}$	*N*

Usually, for convenience, in ***M*** we change the notation of *p* by *k*; also during the statistical analysis, we use the transpose ***M***′*structure* (*k* rows: genes, *p* columns: samples), which shows the samples (e.g. individuals) in the columns and the identified gene in the rows (Table 1).

As a result of genomic analysis, ***M***′ can be very large and usually has thousands of genes, most of them with small frequencies or 0, that is, ***M***′ is typically a sparse matrix. This matrix is truncated because some characteristics were not observed in the sampling.

From the statistical point of view, it is highly convenient to formalize the probability distribution underlying this matrix structure, so each sample from ***M***′ can be represented by one *k*‐dimensional random vector $X_{j};X_{j}=\left(m_{1j},m_{2j},\ldots ,m_{\mathrm{kj}}\right)$, where $m_{\mathrm{kj}}$ represents the number of times that gene *k* is observed in sample *j*.

The probability distribution of each random vector $X_{i.}$ (vector row) and $X_{.j}$ (vector column) can be associated individually with a multinomial distribution,

(1) $$X_{.j}∼MN\left(N_{.j},\theta_{1j},\ldots ,\theta_{\mathrm{kj}}\right);\;\forall j=1,..,n$$

(2) $$X_{i.}∼MN\left(i.,\theta_{i1},\ldots ,\theta_{\mathrm{in}}\right);\;\forall i=1,..,k$$

The multinomial distribution is a multivariate generalization of the binomial distribution, where the marginal distribution of each $X_{\mathrm{ij}}$ is:

(3) $$X_{\mathrm{ij}}∼Bin\left(m_{\mathrm{ij}},\theta_{\mathrm{ij}}\right);\;1\leq \theta_{\mathrm{ij}}\leq 1;\;\forall j=1,..,n;\;\forall i=1,..,k$$

for example, if we consider the partition of all sample space $\Omega^{j}$ the *j*‐sample space in *k* parts:

$$A_{1j},\;A_{2j},\;\ldots \;,A_{\mathrm{kj}}$$

One individual selected randomly has the probability $\theta_{\mathrm{kj}}$ of belonging to the gene $A_{\mathrm{kj}}$ in the partition:

(4) $$\left(\begin{array}{c} P\left(A_{1j}\right)=\theta_{1j}\\ \begin{array}{c} P\left(A_{2j}\right)=\theta_{2j}\\ \vdots \end{array}\\ P\left(A_{\mathrm{kj}}\right)=\theta_{\mathrm{kj}} \end{array}\right\}\sum_{i=1}^{k}\theta_{\mathrm{ij}}=1;\;\forall j=1,..,n$$

If we wish to calculate the probability of sample *j* having $N_{.j}$ individuals, $m_{1j}$ belongs to class $A_{1j}$, $m_{2j}$ to class $A_{2j}$,...,$m_{\mathrm{kj}}$ to class $A_{\mathrm{kj}}$, with the restriction

(5) $$\sum_{i=1}^{k}m_{\mathrm{ij}}=N_{.j};\;\forall j=1,..,n$$

Furthermore, using the multinomial function of density (mass function) we can calculate this probability, $MN\left(N_{.j};\theta_{j}=\left(\theta_{1j},\theta_{2j},\ldots ,\theta_{\mathrm{kj}}\right)\right)$:

(6) $$P\left[\left(A_{1j}=m_{1j}\right)\cap \ldots \cap \left(A_{\mathrm{hj}}=n_{\mathrm{kj}}\right)\right]=\frac{N_{.j}!}{m_{1j}!m_{2j}!\ldots m_{\mathrm{kj}}!}\theta_{1j}^{m_{1j}}\cdot \theta_{2j}^{m_{2j}}\cdot \ldots \cdot \theta_{\mathrm{kj}}^{m_{\mathrm{kj}}};\;\forall j$$

where $0\leq \theta_{\mathrm{ij}}\leq 1$ for all *i* in 1 to *k*, and $\theta_{1j}+\ldots +\theta_{\mathrm{kj}}=1\left(\forall j\right)$, and if *k* = 1, the mass function is reduced to the binomial, ∀j=1,..,n.

The conjugate prior of the Multinomial distribution is the Dirichlet distribution, the multivariate generalization of beta distribution. Hence, the parameter vector $\theta_{k}=\left(\theta_{1j},\theta_{2j},\ldots ,\theta_{\mathrm{kj}}\right)$; ∀j has a prior distribution given by:

(7) $$\theta_{k}∼Dirichlet\left(\alpha_{1j},\alpha_{2j},\ldots ,\alpha_{\mathrm{kj}}\right);\;\forall j=1,..,n$$

In (10), the density function is given by:

$$g\left(\theta |\alpha_{1j},\alpha_{2j},\ldots ,\alpha_{\mathrm{kj}}\right)=\frac{\Gamma \left(\sum_{i}^{k}\alpha_{\mathrm{ij}}\right)}{\prod_{i}^{k}\left(\Gamma \alpha_{\mathrm{ij}}\right)}\theta_{1j}^{\left(\alpha_{1j}‐1\right)}\theta_{2j}^{\left(\alpha_{2j}‐1\right)}\ldots \theta_{\mathrm{kj}}^{\left(\alpha_{\mathrm{kj}}‐1\right)};$$

(8) $$\alpha_{\mathrm{ij}}>0;\;0\leq \theta_{\mathrm{ij}}\leq 1;\;\sum_{i}^{k}\theta_{\mathrm{ij}}=1;\;\forall j=1,..,n$$

In Bayesian inference, $p\left(\theta |x\right)$ is known as posterior distribution and is proportional to likelihood (*p*(*x*|*θ*))*x* prior distribution (*p*(*x*)), so $p\left(\theta |x\right)\propto p\left(x|\theta \right)\cdot p\left(x\right).$

The posterior distribution of $\theta_{j}$ given *X* is:

(9) $$\theta_{j}|x∼Dirichlet\left(x_{1j}+\alpha_{1j},x_{2j}+\alpha_{2j},\ldots ,x_{\mathrm{kj}}+\alpha_{\mathrm{kj}}\right);\;\forall j=1,..,n$$

Thus, in order to implement a new method that calculates the depth of the sample and conveniently estimates the sampling effort, as well as whether it is necessary to sequence more samples or not, matrices ***M***′ can be simulated with different values of *k* and *n*, with ***M***′ requiring a multinomial probability distribution. ***M***′ can be directly simulated from the joint posterior Dirichlet distribution, using the rdirichlet( ) function from the LearnBayes package in R (CRAN, 2018a, 2018b) and the rmultinom( ) function with Dirichlet prior probability (Monleon‐Getino et al., 2019).

### 2.3 Calculating rarefaction curves

There are many methods for calculating the rarefaction curve for each ***M***′; here, we chose to use one of the most recent ones, the iNEXT( ) function of R iNEXT for Interpolation and Extrapolation for Species Diversity (Hsieh et al., 2016). This library provides simple functions to compute and plot two types (sample size‐ and coverage‐based) of rarefaction and extrapolation of species diversity (based on Hill numbers) for individual‐based (abundance) data or sampling unit‐based (incidence) data.

Using the iNEXT( ) function, we calculated the rarefaction curves for each metatranscriptomic/metagenomic matrix (***M***′) simulated previously.

### 2.4 Calculating sampling effort

Unfortunately, iNEXT( ) cannot calculate the maximum number of genes or estimate the sampling effort, and the reads covering 90%, 95%, and 99% of the maximum number of genes in the case of nonsaturated rarefaction curves. To address this caveat, we propose a nonlinear parametric model.

In this type of study, it is common to perform an initial analysis for model selection. Thus, rarefaction curves were first fitted based on previous experience and a selection of possible nonlinear models (Mendez et al., 2017) or the use of Bayesian methods (Monleon‐Getino et al., 2017) were tested.

Several functions, including Weibull, logistic, asymptotic regression through the origin (or a two‐parameter Weibull growth model), Gompertz, and Michaelis–Menten models, were tested using nonlinear regression for use as extrapolations of the rarefaction curves (Mendez et al., 2017). The regression analysis was performed using the R‐package function nls( ), and the model accuracy was tested with the function accuracy( ) of the R‐package rcompanion (R Companion, 2018), which produces a table of statistics that can fit multiple models. The model accuracy was tested using Efron's pseudo r‐squared, Min.max.accuracy (for minimum, maximum accuracy, more substantial indicates a better fit, and a perfect fit is equal to 1), and root‐mean‐square error (RMSE), which has the same units as the predicted values. The Weibull sigmoid model obtained the best scores and was selected as a useful function that fits and extrapolates the rarefaction curve.

The Weibull growth model used in our studies is derived from the one‐parameter Weibull function (10), given by:

(10) $$F\left(x\right)=1‐e^{\left(‐x^{\gamma }\right)}$$

where *γ* is a shape parameter and *x* > 0 and *γ* > 0. The distribution function has a point of inflection at $\left(x,F\left(x\right)\right)=\left(\frac{{\left[\left(\gamma ‐1\right)/\gamma \right]}^{1}}{\gamma },\;1‐\exp\left(‐\left(1‐\gamma^{‐1}\right)\right)\right)$. The following equation can then be used to obtain the sigmoidal curve for empirical use:

(11) $$F\left(x\right)=\beta +\left(\alpha ‐\beta \right)F\left(kx,\theta \right)$$

Moreover, the Weibull function of four parameters can be described by the function $F\left(x\right)=\alpha ‐\left(\alpha ‐\beta \right)e^{‐{\left(\mathrm{kx}\right)}^{\gamma }}.$ Thus, in our case the Weibull growth model of four parameters (Pinheiro, 2018) is described by the function $W\left(x\right)$:

(12) $$W\left(x\right)=a‐be^{‐{\left(\mathrm{cx}\right)}^{m}}$$

where $W\left(x\right)$ is the potential number of genes being expressed for each number of reads (*x*) and now $a=\alpha ,\;b=\alpha ‐\beta ,\;c=\kappa^{\gamma }$ and m=γ. *a*, *b*, *c*, and *m* are parameters to be estimated and *e* is the base of the natural logarithms. *a* is the asymptote of limiting value of the response variable $W\left(x\right)$, $\lim_{x\to \infty }W\left(x\right)=a$, which represents the maximum number of expressed genes. *b* is the biological constant (lower asymptote), *c* is the parameter governing the rate at which the response variable approaches its potential maximum *a*, and finally, *m* is the allometric constant. The four‐parameter Weibull growth model is considered very flexible in that it can be easily transformed into a three‐, two‐, or one‐parameter model to adapt the relation between possible numbers of genes being expressed for each sample size (reads). For example, by setting *b* = *a* and *m* = 1 from (12), we obtained a two‐parameter Weibull growth model (or Asymptotic regression through the origin model given by:

(13) $$W\left(x\right)=a\left(1‐e^{\left(‐cx\right)}\right)$$

with the same meaning W(x), *x*, *a* and *c* (see 12).

### 2.5 Estimation of the amount of sequencing (reads) needed to cover the total expected microbial metatranscriptome/metagenome (confidence band)

The maximum potential number of genes being expressed and the 95% confidence band was used as an estimation of the asymptote of limiting value in a Weibull growth model of four (12) or two parameters (13). Using this Weibull parametric model, we estimated the amount of sequencing needed to cover 90%, 95%, and 99% of the total expected metagenome/metatranscriptome in the samples and the 95% confidence interval, based only on the first 1 million sequences for each sample. We used R (v. 3.6) to perform all the calculations described below.

Parameters in the Weibull growth model were estimated using the nls (Nonlinear regression), nls2 (Nonlinear regression with brute force (CRAN, 2018b), and minpack.lm (R Interface to the Levenberg‐Marquardt nonlinear least squares) packages. The option ~ *Ssweibull(x; a*, *b*, *c*, *m)* was used for the four‐parameter Weibull growth model, and ~ *SsasympOrig(x; a*, *b)* was used for the two‐parameter Weibull model. In order to initialize the parameters, a "brute‐force" algorithm was used, and then, the parameters were optimized until those that maximize the adjustment value were optimized; the "brute‐force" algorithm returns the nls object corresponding to the starting values (CRAN, 2018b).

### 2.6 A priori gene prediction using a few initial total reads

We used different algorithms to fit a regression model to predict the potential number of genes, effort/reads covering 90%, 95%, or 99% of the maximum number of genes based on the first 10%–20% of sequences (reads). As a first strategy, a classical linear regression of the function lm( ) was optimized using a function step( ) to perform the stepwise model selection, and the model was validated using the function cv.lm (data, model, m) from the DAAG library (Maindonald & Braun, 2010, 2019). This function gives internal and cross‐validation measures of predictive accuracy for multiple linear regression.

Two other strategies were applied: the so‐called machine learning algorithms such as support vector machines (SVM) and Extreme Gradient Boosting (XGBoost), in which we used the training data (with multiple features)* x_i_* (here the genes in each sequencing depth) to predict a target variable *y_i_* (maximum number of genes).

Support vector machines (SVM) constitute a data classification method that separates data using hyperplanes, which is useful in the case of regression (Cortes & Vapnik, 1995). If we have labeled data, SVM can generate multiple separating hyperplanes, so that the data space is divided into segments, each containing only one kind of data. The SVM technique is generally useful for data which has nonregularity, that is, without a known distribution. We used the function SVM( ) in R for the calculation (Chang & Lin, 2011).

Extreme Gradient Boosting is an efficient implementation of the gradient boosting framework from Chen and Guestrin (2016). Gradient boosting is a state‐of‐the‐art prediction technique that sequentially produces a model in the form of linear combinations of simple predictors—typically decision trees—by solving an infinite‐dimensional convex optimization problem. XBoost( ) from library Xboost( ) in R (Chen & Guestrin, 2016) permits the calculation of this predicted method.

In order to check the accuracy of different models, it is common to use the coefficient of determination (*R* ^2^ or R‐squared), the mean absolute error (MAE), and the root‐mean‐square error (RMSE) (Hyndman & Koehler, 2006).

*R* ^2^ is the percentage of the response variable variation that is explained by the model:

(14) $$R^{2}=\text{Explained}\;\text{variation}/\text{Total}\;\text{variation}$$

*R^2^* is always between 0 and 1, 0 indicating that the model explains none of the variability of the response data around its mean and 1 that the model explains all the variability of the response data around its mean.

RMSE is a frequently used measure of the differences between values (sample and population values) predicted by a model or an estimator and the values observed. The RMSE represents the sample standard deviation of the differences between predicted and observed values.

(15) $$\text{RMSE}=\frac{\sum_{i=1}^{n}{\left(\hat{y_{i}}‐y_{i}\right)}^{2}}{n}$$

where *n* is the number of pairs of observations, $\hat{y_{i}}$ the value predicted and *y_i_* the observed value.

Mean absolute error (MAE) is the average vertical distance between each point and the *Y = X* line:

(16) $$\text{MAE}=\frac{\sum_{i=1}^{n}\left|\left(\hat{y_{i}}‐y_{i}\right)\right|}{n}$$

where *n* is the number of pairs of observations, $\hat{y_{i}}$ the predicted value and *y_i_* the observed value.

### 2.7 Metatranscriptome databases used in the method application

We used metatranscriptome datasets from three different sources for the application of the proposed method. The first set was generated in our lab as described in Yost et al. (2015) and is available at the Human Oral Microbiome Database (HOMD) server under the submission number 20141024 (ftp://ftp.homd.org/publication_data/20141024/RNA/ ). The second dataset was generated by Benítez‐Páez et al. (2014) and is available at the MG‐RAST server by accessing the “Oral Metatranscriptome” project, id 935 (http://metagenomics.anl.gov/linkin.cgi?project=935). The third dataset was generated by Jorth et al. (2014) and is available at DNAnexus study number SRP033605 (http://sra.dnanexus.com/studies/SRP033605). All databases were bioinformatically cleaned of rRNA sequences, and in the case of SRP033605, we also removed low‐quality sequences from the query files. Fast clipper and fastq quality filters from the Fastx toolkit (http://hannonlab.cshl.edu/fastxtoolkit/) were used to remove sequences shorter than 50 bp with a quality score > 20 in > 80% of the sequence.

## 3 RESULTS AND DISCUSSION

### 3.1 Metatranscriptomic/ Metagenomic matrix simulation, rarefaction computation, and estimation of parameters

Our focus was to study the transcriptome of whole complex microbial communities rather than individual transcriptomes, using an oral community as a model. The oral microbiome is one of the best characterized human body sites (Belda‐Ferre et al., 2012; Haffajee et al., 2008; Marsh, 2006; Paster et al., 2001; Peterson et al., 2013; Socransky et al., 1998), comprising an extremely complex and highly organized biofilm community (Kolenbrander, 2000; Kolenbrander et al., 2002). More than 700 bacterial species have been identified in the oral cavity [Paster et al., 2001; Dewhirst et al., 2010]. Many oral bacterial species have not yet been cultivated, and the only information we possess about them derives from their 16S rRNA phylogenetic affiliation.

In the current study, we investigated the proposed mathematical Weibull model, using nonlinear regression modeling. This model is a generalization of the asymptotic growth model in that it reduces when the parameter m is unity (see Methods).

Using an R script (see Supplementary Material), we simulated 1,587 metatranscriptomic/metagenomic matrices containing more than $9^{9}$ reads, with random numbers of genes (min = 267, max = 339,319) and reads (min = 550, max = 6,823,774), and always 3 samples (replicas). The simulations had a high computational cost of more than 2 weeks and were carried out on a Linux Xeon SP 4114 2.2 GHz computer server with 40 cores. This information has been collected in a data frame for further analysis.

A rarefaction curve using the 1,587 simulated cases was computed using the function iNEXT( ), and the vector obtained (*n* = 100 points, *x* = reads, *y* = genes) was saved and used later to compute (a) the maximum number of genes, (b) the sampling effort to reach the maximum number of genes (minimum = 1%, maximum = 100%; see Table 2), and c) the reads covering 90, 95 and 99% of the maximum number of genes. This last part [points (a), (b), and (c) ] was done using an estimation based on the Weibull model described in Section 2.3 using nonlinear regression.

**TABLE 2:** Estimations of parameters of interest using a set of 1,587 simulations by means of a multinomial model of a metatranscriptomic/ metagenomic matrix

Estimations of parameters of interest	Mean	Minimum	Maximum
Maximum number of genes observed	183,312	6	926,470
Effort computed using the iNEXT( )+Weibull model	72.399%	1.208%	100%
Reads covering 99% of the maximum number of genes	5,788,494	80	31,779,350

**FIGURE 1:**
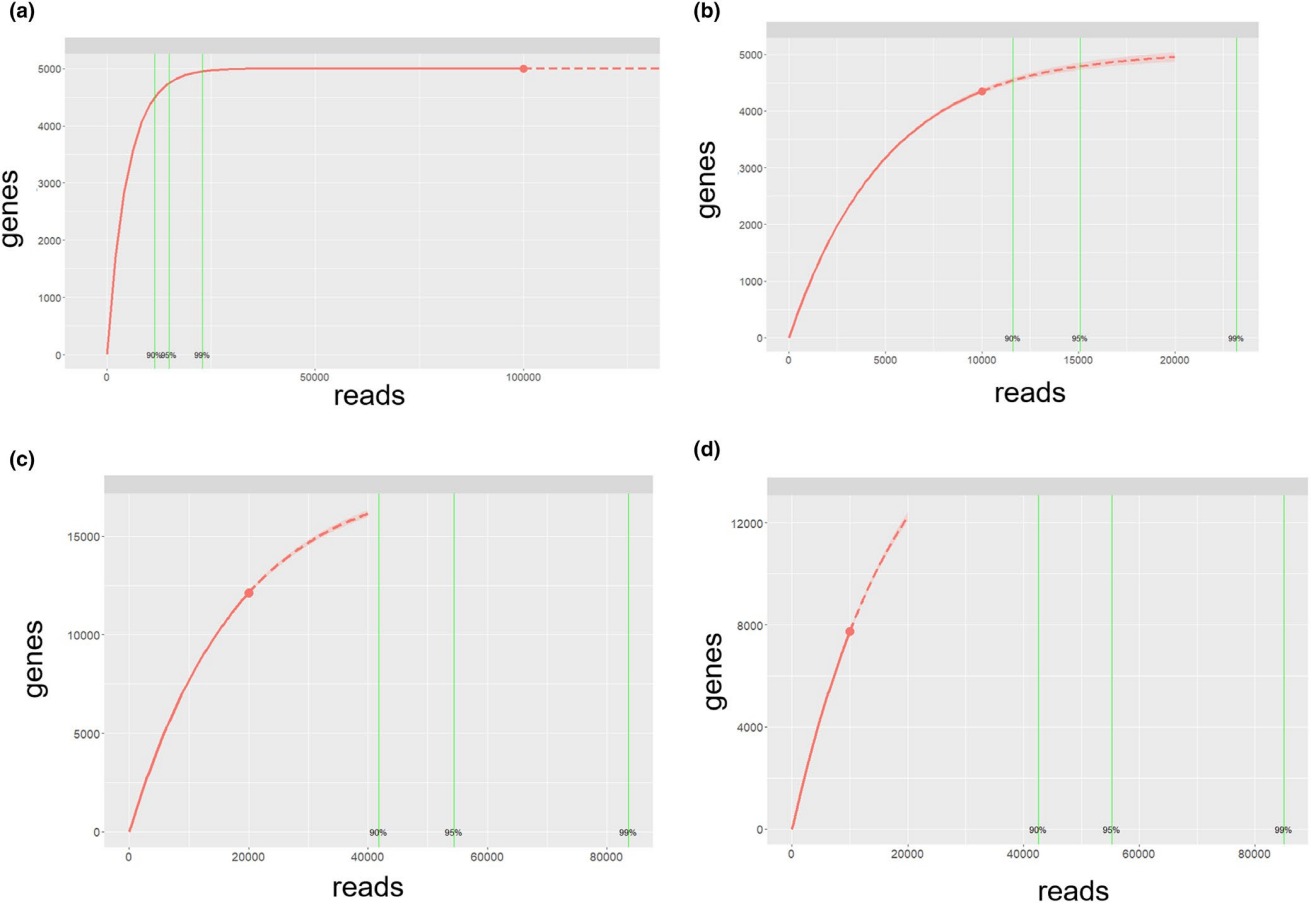
Calculation of the number of genes versus the number of reads using the PILI3( ) function of the library(Sequencingeffort). Interpolated data (solid red line), extrapolated data (dashed line), and red dot (limit of observed data)

Four examples of the results obtained are shown in Figure 1. The results can be distinguished in four different types of rarefaction curves:

- Over‐sampling curves: minimum sampling effort to obtain the maximum amount of genes in a quick rarefaction curve (Figure 1.a).
- Correct sampling curves: medium sampling effort to obtain the maximum amount of genes in a saturated rarefaction curve (Figure 1.b).
- Under‐sampling curves: maximum sampling effort to obtain the maximum amount of genes in a nonobserved saturated rarefaction curve (Figure 1.c).
- Very under‐sampling: very maximum sampling effort to obtain the maximum amount of genes in a nonobserved saturated rarefaction curve (Figure 1.d).

Moreover, in the curves of Figure 1, we can distinguish the vertical lines of the reads covering 90%, 95%, and 99% of the maximum number of genes.

### 3.2 A priori gene prediction using only a few total reads

Using the simulated data and the parameters estimated previously, we fitted a regression to predict the potential number of genes and the reads covering 90%, 95%, or 99% of the maximum number of genes using only the first 20% of sequences (reads). To implement this method, we used three algorithms (linear model (lm), Extreme Gradient Boosting (XB), and support vector machine (SVM)) to predict the aforementioned values. Several predictors were tested to predict the maximum number of genes as a function of the first 20% of sequences (reads). Using the simulated data, several good predictors were detected, such as the asymptote, using a four‐parameter Weibull model or other similar and well‐known models such as the logistics curve model (Mendez et al., 2017). Other predictors used were the minimum–maximum number of genes observed and, finally, the minimum–maximum number of reads observed (see Table 3, central column; model 1 and model 2 and supplementary material).

**TABLE 3:** Model accuracy for the prediction of the maximum number of genes using only 20% of total reads in a simulation of 1,587 metatranscriptomic/metagenomic genomic sequences

Model name	Predictors used in the model (independent variables, *X_i_*)	Results (*R* ^2^) with different algorithms of prediction
Model 1	Asymptote estimated using a logistic function Asymptote estimated using a four‐parameter Weibull function “Observed” minimum number of reads of the 20% vector “Observed” maximum number of reads of the 20% vector	SVM = 0.9964754 LM = 0.9990069 Xboost = 0.999999
Model 2	Asymptote estimated using a four‐parameter Weibull function “Observed” minimum number of reads of the 20% vector “Observed” maximum number of reads of the 20% vector	SVM = 0.9964423 LM = 0.9981882 Xboost 0.9999981

After testing the prediction of the proposed models using the three aforementioned prediction algorithms (lm, Xboost and SVM), it was found that the results of the prediction of interest (maximum number of genes and reads covering 90%, 95%, or 99% of the maximum number of genes) for the total curve with the 1,587 simulated samples were very similar, with an *R* ^2^ > 0.99, which indicates a possible over‐fitting (see Table 3, right column).

To validate the method and the models, we initially used only the first 20 points of the rarefaction curve (reads of 20% of the total amount of the curve obtained) and then divided the total number of simulated rarefaction curves (*n* = 1,587) and the estimated parameters (maximum number of genes, sampling effort, etc.) into two parts using cross‐validation: (a) In the training set, 70% was used to train and estimate the prediction models (lm, XB, and SVM), and (b) in the test set, 30% was used to check the model fit and capacity to predict the maximum number of genes, and reads covering 90%, 95%, and 99% of the maximum number of genes using only the first 20% of sequences (reads).

We used 300 random resamplings, and a significant computational effort was made to obtain the predictions using models 1 and 2. We determined that the XB and lm are useful methods to predict the maximum number of genes using only 20% of sequencing depth. To prove the accuracy of the method, we used the mean absolute error (MAE), root‐square‐mean error (RSME), and the coefficient of determination (*R* ^2^) between estimations using the Weibull model with 100% and 20% of the rarefaction curve.

**FIGURE 2:**
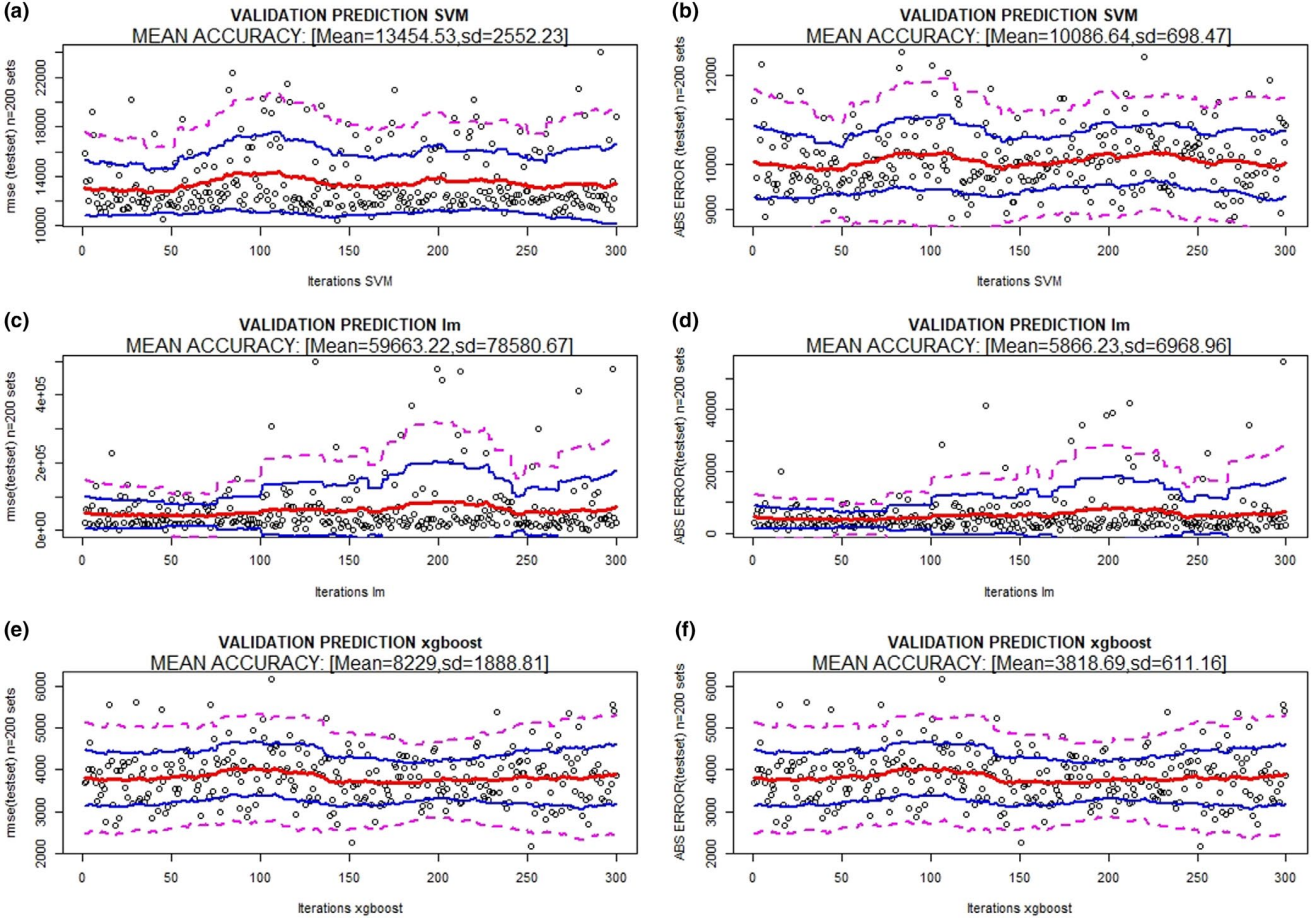
RMSE and absolute error bands (mean (red), 95% (blue), and 99% confidence (magenta)) of different methods [(a) support vector machine, (b) linear regression model, and (c) XBoost] using 20% of sequencing depth (reads) to predict the maximum number of genes. 300 random resamples were performed

**FIGURE 3:**
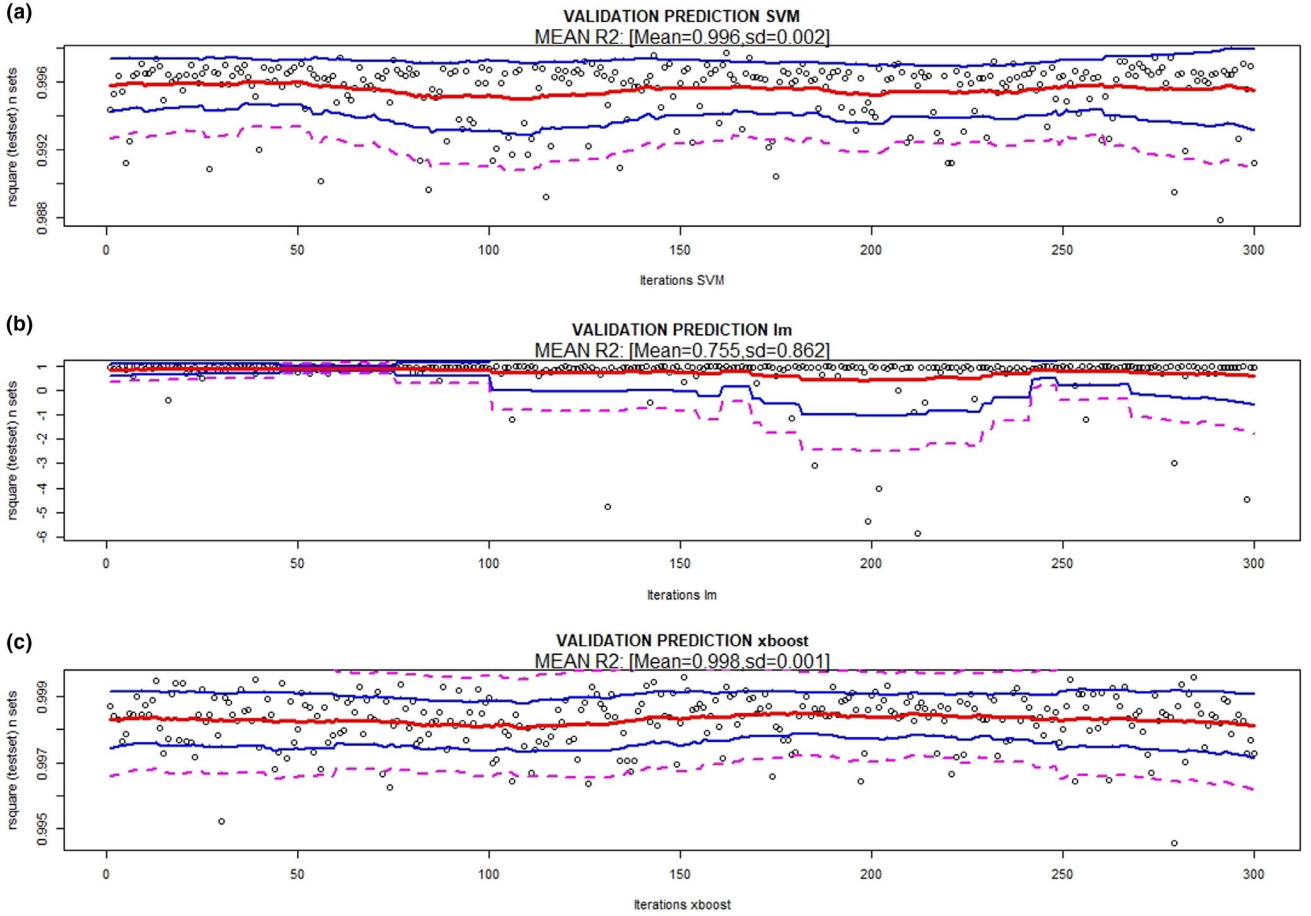
Coefficient of determination (*R* ^2^) bands (mean (red), 95% (blue) and 99% confidence (magenta)) of the different methods used [(a) Support vector machine, (b) linear regression model, and (c) XBoost] using 20% of sequencing depth (reads) to predict the maximum number of genes. 300 random resamples were performed

The results of the validations of the three prediction methods (XB, lm, and SVM) and model 1 are presented in Figures 2 and 3 (prediction of maximum number of genes) and 4 and 5 (prediction of reads covering 95% of the maximum number of genes), which show the absolute error (MAE), RMSE bands (mean and 95% and 99% confidence), and *R* ^2^ for the 300 random resampling test sets. It can be observed that SVM and XB are the best methods in all situations (estimation of the maximum number of genes; number of reads to cover 95% of the maximum number of genes).

**FIGURE 4:**
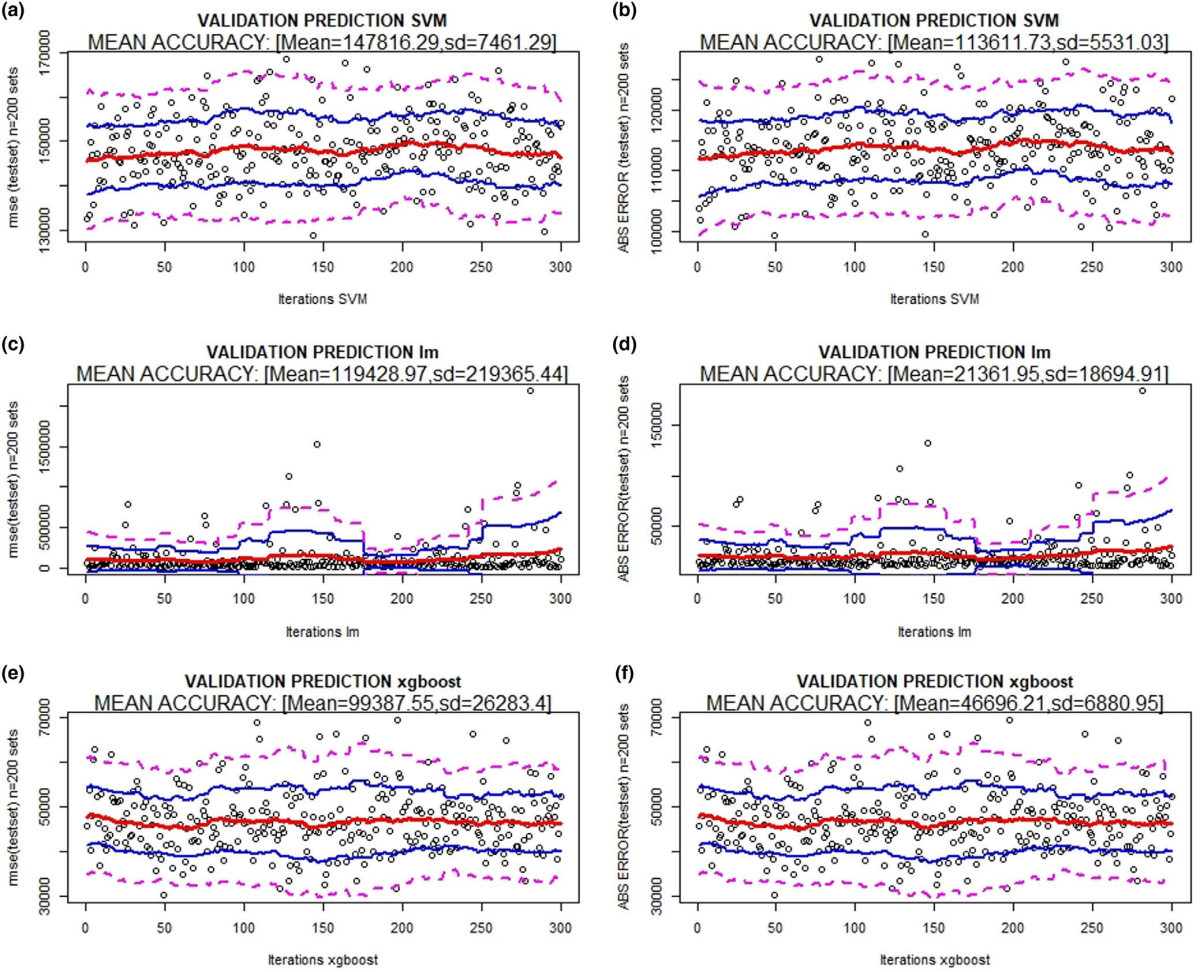
RMSE and absolute error bands (mean (red), 95% (blue), and 99% confidence (magenta)) of different methods [(a) Support vector machine, (b) linear regression model, and (c) XBoost] using 20% of sequencing depth (reads) to predict the reads covering 95% of the maximum number of genes. 300 random resamples were performed

**FIGURE 5:**
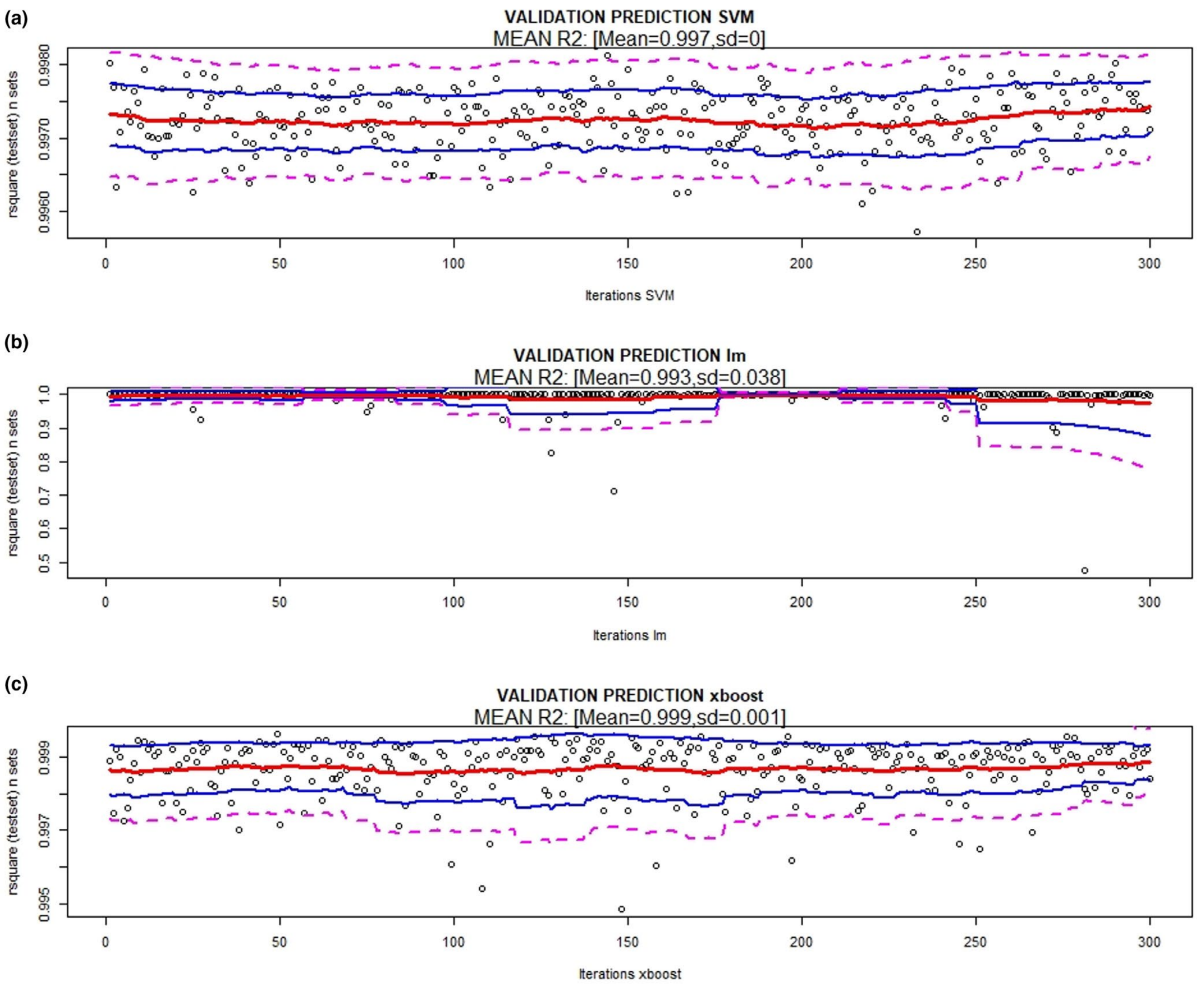
Coefficient of determination (*R* ^2^) bands (mean (red), 95% (blue), and 99% confidence (magenta)) of the different methods used. [(a) Support vector machine, (b) linear regression model, and (c) XBoost] using 20% of sequencing depth (reads) to predict the reads covering 95% of the maximum number of genes. 300 random resamples were performed

The final SVM method (model 1) for predicting the maximum number of genes has an RMSE = 13,454, MAE = 10,086 and *R* ^2^ = 0.996 between the observed and predicted values (Figure 2(a,b) and Figure 3(a). The final SVM model (model 1) for predicting reads to cover 95% of the maximum number of genes has an RMSE = 147,816, MAE = 113,611 and *R* ^2^ = 0.997 between the observed and predicted values (Figure 4(a,b) and Figure 5(b).

The final XB model estimated to predict the maximum number of genes has an RMSE = 8,229, MAE = 3,819 and *R* ^2^ = 0.998 between the observed and predicted values (Figure 2(e,f) and Figure 3(c). The final XB model estimated to predict reads covering 95% of the maximum number of genes has an RMSE = 99,388, MAE = 46,696 and *R* ^2^ = 0.999 between the observed and predicted values (Figure 4(e,f) and Figure 5(c).

**FIGURE 6:**
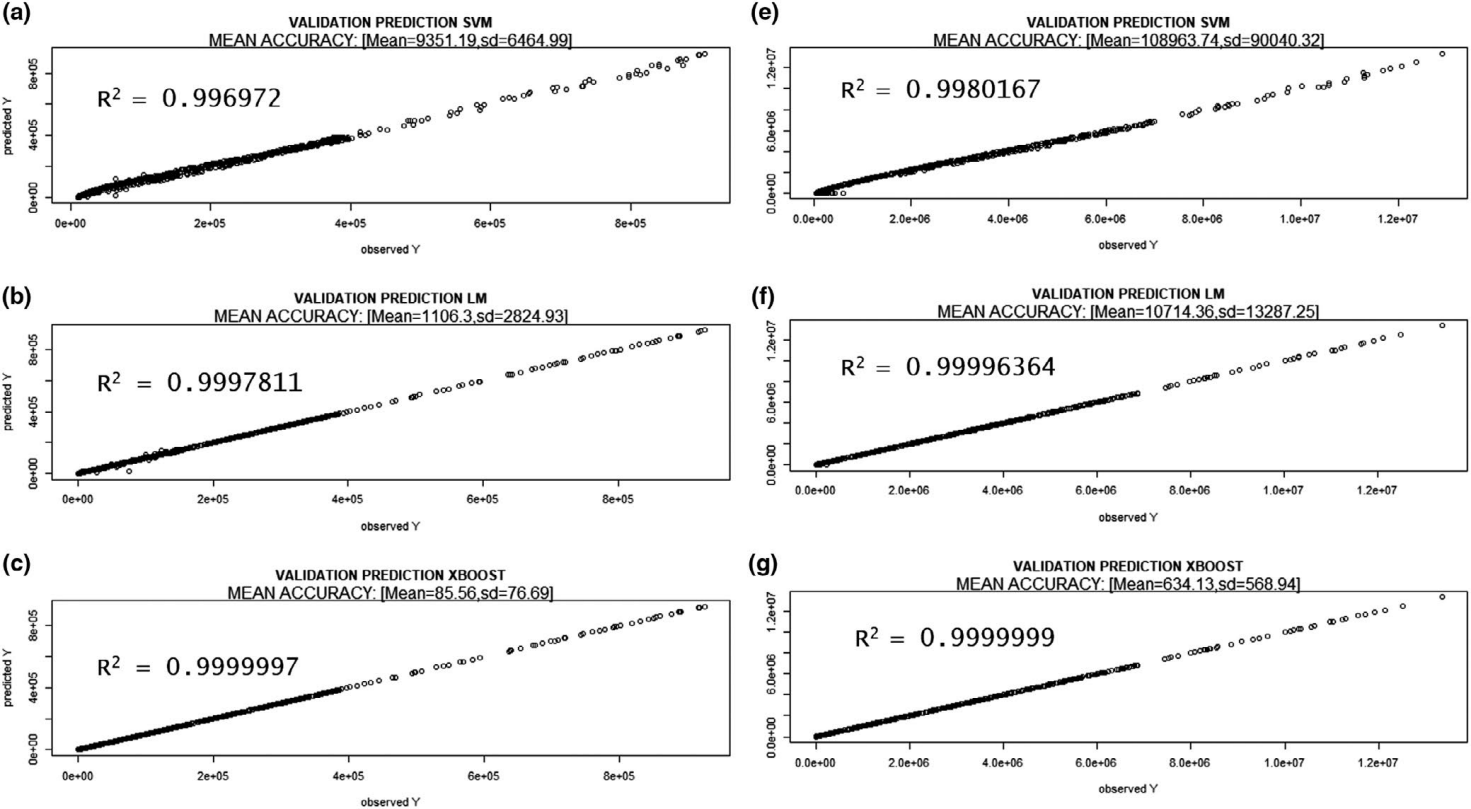
Prediction of maximum number of genes (a, b, c) and reads covering 95% of the maximum number of genes (d, e, f) using a SVM, lm, and XGBoost model and only 20% of the reads versus the observed value. All the samples were used (*n* = 1,556)

Finally, an XB model 1 including the total amount of simulated data (*n* = 1,587) was estimated and saved. The *R* ^2^ of all data and prediction models (lm, XB, and SVM) are presented in Figure 6. This model will be used to predict the described parameters of interest (maximum number of genes: Figure 6 a,b,c, reads to cover 95% of the maximum number of genes: Figure 6d,e,f, effort, etc.). Also, the confidence interval (95%) of the prediction was obtained by applying a "bagging" method, which was possible with the XB model and involves creating the same model many times (with randomness).

Finally, using 100 subsamples we obtained the prediction mean and 95% by means of the function ci.mean( ) of the library(Publish) for R. The final XB model estimated to predict the maximum number of genes has an MAE = 86 and *R* ^2^ = 0.9999997 between the observed and predicted values (Figure 6c). The final XB model estimated to predict reads covering 95% of the maximum number of genes has an MAE = 634 and *R* ^2^ = 0.9999999 between the observed and predicted values (Figure 6g).

### 3.3 Application of the proposed method to real data

External validation (real data not used before) was performed to check the algorithms developed previously. To this end, we used a set of 15 datasets of metatranscriptomes from the oral cavity. These RNA sequences consist of vectors of 10^5^–1.5 × 10^7^ read depth with a 10,000 and 600,000 gene size, most of them with saturation but in some cases with a definite unsaturation. We used these sequences to validate the method and predict the maximum number of genes and the number of reads covering 95% of the maximum number of genes, using all the available reads or only a percentage (3%, 20%, and 60% of read depth). The function monle.predict.max( ) was developed to compute this type of incomplete transcriptomic vectors (*X* = sequencing depth, *Y* = genes).

The results of this validation are shown in the supplementary material and reflect that the used model, based on a four‐parameter Weibull model, had a perfect fit and could correctly estimate the parameters of interest (maximum number of genes, read depth covering 95% of the maximum number of genes).

When only a percentage (3%, 20%, and 60% of read depth) of the transcriptomic vector was used, the results were also quite acceptable for predicting the maximum amount of genes and moderately acceptable for predicting the reads covering 95% of the maximum number of genes. The prediction for the maximum number of genes was considered acceptable when the maximum number of genes was within the XB bagging 95% prediction interval. Similarly, the prediction of the read depth covering 95% of the maximum number of genes was considered acceptable when within the XB bagging 95% prediction interval or between the 90%–99% interval calculated using the 100% read depth of the transcriptome.

When 3% (10^5^–5 × 10^5^ reads) was used to predict the parameters of interest, 12/15 (80%) curves to predict the number of genes and 6/15 (33%) curves to predict the reads covering 95% of the maximum number of genes were acceptable. When 20% (105–3 × 106 reads) was used to predict the parameters of interest, 14/15 (93%) curves to predict the number of genes and 9/15 (60%) curves to predict reads covering 95% of the maximum number of genes were acceptable. When 60% (105–1 × 107 reads) were used to predict the parameters of interest, 14/15 (90%) curves to predict the number of genes and 9/15 (60%) curves to predict reads covering 95% of the maximum number of genes were acceptable.

### 3.4 Conclusions

This proposed method to estimate the maximum number of genes and the reads covering 90, 95, and 99% of the maximum number of genes, using an algorithm based on a rarefaction curve + Weibull model + machine learning prediction, will help researchers to know whether sampling is sufficient or needs to be increased. The method should be used with precaution when predicting the sequencing depth, especially with unsaturated samples. However, although the proposed model can cause predictive problems, it was found to work in most cases. Further studies using real sequences and typologies should be carried out to fully validate the model and the simulation‐based methodology.

Estimating the sequencing depth required to adequately sample the target metatranscriptome/metagenome using RNA‐seq, and Shotgun is an essential first step in obtaining robust results in subsequent analysis and avoiding overexpansion once the information contained in the library reaches saturation. Our method allows the use of an initial shallowly sequenced sample (in this case 20% of the total amount of reads sampled) to estimate the sequencing effort needed to cover the whole metatranscriptome/metagenome from the same sample and therefore to estimate the sample size. The initial number of sequences is low enough for current NGS methods to analyze a considerable number of samples at a low cost.

## CONFLICT OF INTEREST

The authors declare no conflict of interest.

## AUTHOR CONTRIBUTIONS

**Toni Monleon‐Getino:** Conceptualization (equal); data curation (lead); formal analysis (lead); investigation (equal); methodology (equal); software (equal); writing – original draft (equal); writing – review and editing (equal). **Jorge Frias‐Lopez:** Conceptualization (equal); methodology (equal); writing – original draft (lead); writing – review and editing (lead).

## Supporting information

Supplementary Material

## Data Availability

All code is open source and available in Github. All functionalities shown in Figure 1 (rarefaction curve, Weibull nonlinear model, effort estimation, extrapolation of the maximum number of genes, reads covering 90, 95, and 99% of the maximum number of genes) have been compiled in two new functions in R: PILI3( ) and monle.predict.max( ) and added to the library**(**Sequencingeffort) and can be found at the repository https://github.com/amonleong/Sequencingeffort. For each described function, at least two examples of use are given, as well as the explanation of the arguments of the functions.
