# Supplementary material for: A priori estimation of sequencing effort in complex microbial metatranscriptomes
Source: Ecol Evol. 2020 Nov 5;10(23):13382–94. doi: 10.1002/ece3.6941 (PMC7713979; doi:10.1002/ece3.6941)
Supplement: Supplementary file 1 — Supplementary Material [file ECE3-10-13382-s001.pdf]

Supplementary material -

Simulation of a Metatranscriptomic/metagenomic matrix (R script)

```
nsites<-round(runif(1, min = 500, max = 50000),0)#NUMBER OF GENES
  nsimulac<-3 #NUMBER OF REPLICATIONS
  ab.total<-round(runif(1, min = 3000, max = 300000),0) #abundance
  library(LearnBayes)
  ppp <- rdirichlet(1, par = rep(1, nsites))
  matriu <- array(0, dim=c(nsites, nsimulac)) # MATRIX SIMULATED
  for(i in 1:nsimulac){
    X <- as.vector(rmultinom(1, size =ab.total , prob = ppp))
    matriu[,i]<-X
    N <- sum(X)
  }
```
